# Diagnostic Approach to Hypersensitivity Pneumonitis: A Report of Two Cases

**DOI:** 10.7759/cureus.43290

**Published:** 2023-08-10

**Authors:** Chaynez Rachid, Meryem Hindi, Oussama Fikri, Lamyae Amro

**Affiliations:** 1 Pneumology Department, Chu Mohammed VI, Laboratoire de Recherche Morpho Sciences, Faculté de médecine et de Pharmacie de Marrakech, Université Cadi Ayyad (Labo LRMS, FMPM, UCA) Hopital Arrazi, Marrakesh, MAR

**Keywords:** fibrosis, mosaic lung, bird breeder's lung, farmer's lung, hypersensitivity pneumopathy

## Abstract

Hypersensitivity pneumonitis (HP) is a bronchopulmonary granulomatosis of immunological mechanism, caused by an aberrant response to an inhaled exposure, which results in mostly T cell-mediated inflammation, granuloma formation, and fibrosis in some cases. The most common forms are bird breeder's disease and farmer's lung disease. The diagnosis of HP is based on the presence of compatible symptoms, the notion of exposure to antigens known to be pathogenic, and the existence of interstitial and bronchiolar images on the thoracic scan, lymphocytosis in the alveolar lavage, and precipitins. Chronic forms, in case of insidious exposure, especially in poultry, may evolve into pulmonary fibrosis with a poor prognosis. Through this work, we want to underline the frequency of this disease in our country, its heterogeneity as well as the difficult early diagnosis. Finally, we will investigate the therapeutic effect of corticosteroids in the early stages and the antifibrotic treatment in fibrotic forms.

## Introduction

Hypersensitivity pneumonitis (HP) is a lung disease caused by an immunological response to inhaled organic antigens or other particles. Extrinsic allergic alveolitis is another name for HP. Coughing, shortness of breath, chest pain, and exhaustion are some of the symptoms. There are two types of HP: fibrotic and non-fibrotic. The severity of symptoms varies according to the kind of HP, the amount of triggering agent exposure, and the individual's immune system response. A combination of clinical symptoms, exposure history, and imaging or lung function testing is usually used to make the diagnosis of HP. Treatment may include avoiding further contact with moldy dust or the triggered stimulus, as well as administering corticosteroids or other immunosuppressive medications [1].

Our goal in this article is to summarize the management of fibrotic and non-fibrotic HP by describing our experience with two cases to promote prompt diagnosis and management. 

## Case presentation

Patients with known hypersensitivity lung disease antigen exposure, lymphocytosis in bronchoalveolar lavage (BAL), radiological appearance compatible with hypersensitivity lung disease on high-resolution CT, and positive precipitin levels were included.

Case 1

**Figure 1:**
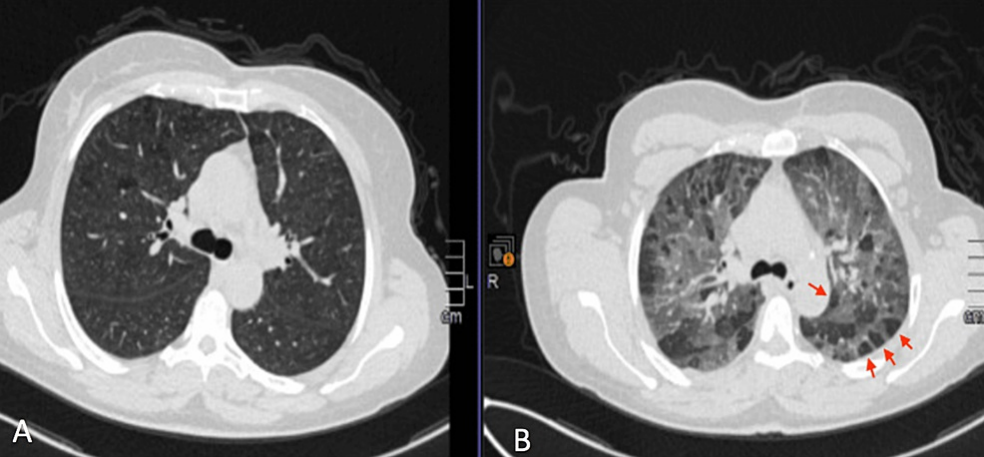
Chest CT scan in the parenchymal window showing a mosaic aspect. High-resolution chest CT scan sections in inspiration (A) and forced expiration (B) with MINI IP reconstruction showing a mosaic aspect with expiratory trapping (>5 lobules per lobe) and a "head cheese" aspect.

**Figure 2:**
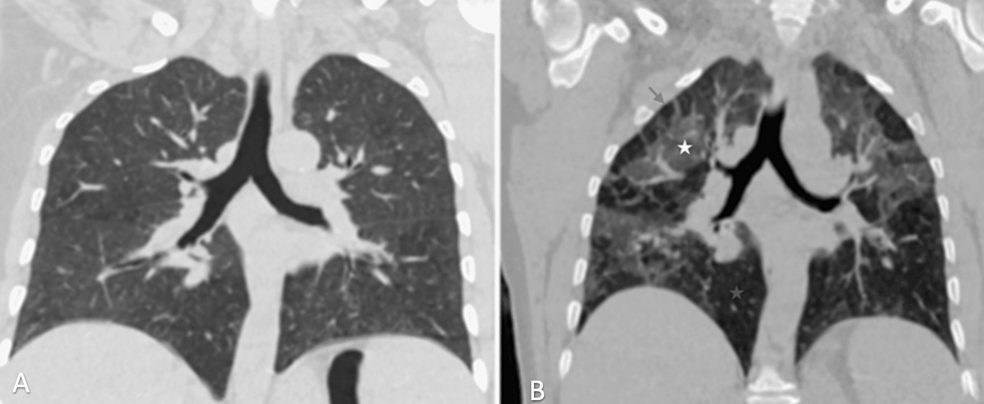
Chest CT scan in the parenchymal window showing an appearance of three densities. Chest CT scan in the axial window in inspiration (A) and forced expiration (B) showing healthy parenchyma, air trapping, and increased ground glass.

A 59-year-old female poultry farmer, nonsmoking, has a history of asthma. The patient presented to the clinic with dyspnea, dry cough, and fatigue that had persisted for four months. She reported that her symptoms worsened after exposure to poultry droppings in the workplace. The clinical examination revealed an asthenic patient with accessory muscle respiration. Lung auscultation revealed crackles. High-resolution axial and coronal slices during inspiration and forced expiration, visualized by MINI IP reconstruction, showed a mosaic aspect with expiratory retention (Figure 1) and a "cheese head" aspect or three densities (Figure 2).

BAL was performed, which revealed pleomorphic inflammatory cytology, predominantly lymphocytic alveolitis 30%, 20% of neutrophils (PNN), 5% of eosinophil, and 45% of macrophages. Determination of serum precipitin included immunological characteristics of breeding bird lungs, anti-pigeon serum antibodies >200 mg/L in two precipitation arcs, anti-feces antibodies in three precipitation arcs, and 15.5 mg/L anti-chicken serum antibodies. Diagnosis is based on the patient's medical history, physical examination, and immunological tests. A diagnosis of farmer's lung disease was made. Spirometry during the diagnosis process revealed severe restrictive respiratory impairment with a forced vital capacity (FVC) of 40%, total lung capacity of 46%, and diffusing capacity of the lungs for carbon monoxide of 31%. 

The patient was advised to avoid exposure to pigeons and was started on corticosteroids. After three months of corticosteroid therapy at a dose of 40 mg per day and scannographic improvement, the patient's symptoms improved with treatment and she was able to return to her normal activities. Follow-up pulmonary function tests showed improvement in lung volumes with an FVC of 48% and diffusing capacity of the lungs for carbon monoxide of 35%.

Case 2 

**Figure 3:**
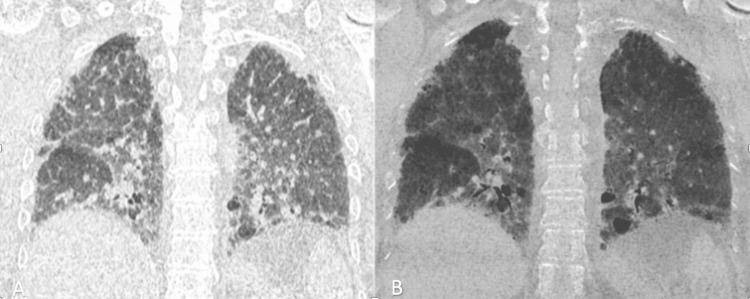
High-resolution chest CT scan in coronal slices in inspiration and expiration showing fibrosis with a mosaic aspect. High-resolution chest CT scan in axial and coronal slices in inspiration (A) and expiration (B) with MINI IP reconstruction showing a mosaic aspect with expiratory trapping, with individualization of cystic lesions of random distribution, subpleural reticulations, and traction bronchiectasis testifying to the fibrosing involvement.

A 56-year-old female patient with a history of progressive dyspnea and dry cough was referred to the pulmonary unit for further evaluation. The patient had been exposed to organic dust in the workplace for the past 20 years as a farmer. On physical examination, it was found that the breath sounds of the patient's lungs were diminished, with inspiratory crackles. A chest CT scan revealed a diffuse interstitial pattern consistent with fibrosis (Figure 3). Spirometry results revealed a restrictive pattern, with an FVC of 45% and diffusing capacity of the lungs for carbon monoxide of 39%. Antibodies against farmer's lung (screening) were confirmed by immunoelectrophoresis, and BAL and transbronchial biopsy were performed to make a definitive diagnosis. The BAL fluid analysis revealed an elevated number of 38% lymphocytes and 40% macrophages, consistent with interstitial inflammation. 

The transbronchial biopsy showed fibrotic changes in the alveolar septa, leading to an opinion of fibrosing HP. The patient was advised to avoid exposure to organic dust and was started on 40 mg of corticosteroids and was also given 4 L/min supplemental oxygen to manage her hypoxemia. Over the ensuing many months, the patient showed improvement in her symptoms and improved values of FVC from 45% to 48% with an increase in FEV_1 _(forced expiratory volume in 1 second) to 80% of the predicted value and a slight enhancement in the follow-up CT.

The patient was followed up regularly over the next two years, and her condition remained stable, with no evidence of progression of fibrosis. She remained on corticosteroids and supplemental oxygen, and her symptoms were well controlled.

## Discussion

HP is a complex interstitial lung disease caused by inhaled antigens. It is a rare but varied form of inflammatory interstitial lung disease caused by the inhalation of antigens with a diameter of less than 5 μm, which can reach the alveoli. These antigens may be bacteria, fungi, or animal proteins such as those in the droppings or feathers of certain birds. The bacteria concerned are mainly thermophilic actinomycetes or *Mycobacterium avium*, found in heated areas such as jacuzzis. *Penicillium* spp. and *Aspergillus *are the fungi species most frequently associated with the onset of disease. If left untreated, HP can lead to progressive pulmonary fibrosis, with its associated morbidity and mortality [2].

The ATS/JRS/ALAT Committee defined HP, and clinical, radiographic, and pathological features were described. In addition to providing algorithms for scoring patients with suspected HP, these guidelines use clinical, radiographic, and pathologic findings to identify fibrotic or non-fibrotic HP. Triage of patients is recommended [3]. 

The diagnosis of HP can be relatively difficult to establish due to the absence of standardized, validated diagnostic criteria. Recent consensus guidelines list important factors supporting the diagnosis, including the history of exposure, confirmatory CT scan, BAL revealing the presence of lymphocytosis, histopathological signs, malformed granulomas, cellular interstitial pneumonia, bronchiolitis, and exposed antigen-specific immunoglobulin G serology [4]. According to expert committees, when high-resolution CT findings are suggestive of HP and antigen exposure is known (from patient history or laboratory tests), lymphocytosis revealed by BAL is sufficient to establish the diagnosis with a high degree of certainty. However, in its absence, a lung biopsy must be performed to establish a precise diagnosis of HP [5].

In our case report, we report two cases of HP in their fibrosing and non-fibrosing forms. The results of chest radiographs and CT scans change depending on the stage of HP. It is preferable to distinguish radiologically between fibrotic and non-fibrotic HP.

In non-fibrotic forms of HP, centrilobular nodules, air trapping, and ground glass opacity, occasionally with nodules or reticulonodular opacities are found. Reticular opacities and honeycombing are the symptoms of chronic HP. The basic management strategy in HP is to identify antigen exposure and fully avoid the problematic antigen (recommended). Corticosteroids are given in situations when there is no remission with antigen avoidance [6]. Some patients previously diagnosed with certain types of fibrotic interstitial lung diseases (f-ILDs), including fibrotic HP (f-HP), are susceptible to developing a progressive fibrosing phenotype (PF-ILD), despite initial state-of-the-art management. Once fibrosis develops in chronic HP patients, it is irreversible and may lead in severe cases to pulmonary transplantation.

To characterize and assess the prevalence of PF-ILD criteria in patients with a multidisciplinary diagnosis (MTD) of chronic f-HP, a cohort study was performed in a Portuguese hospital. Eighty-three individuals with an MTD of HP had been observed for at least a year. Sixty-three (75.9%) of these were diagnosed with f-HP. The predefined criteria for PF-HP were met by 33.3% (n = 21) of the 63 f-HP patients: 66.7% had a relative decline of 10% FVC; 5% had a relative decline of 59% FVC, with worsening symptoms or increased fibrosis seen on high-resolution CT scan; and 23.8% had worsening respiratory symptoms with radiological progression.

This single-center cohort study demonstrated that one-third of the f-HP patients with PF-ILD assessed progression during standard primary care. A common form of interstitial pneumonia, usual interstitial pneumonia (UIP)/UIP-like, was present in >70% of patients with f-HP, and two-thirds of these patients had a decrease in FVC of ≥10%. Patients with PF-HP are also more likely to have exacerbations. Based on the current study data, this group of patients can be considered viable candidates for anti-vibration therapy with a reasonable prospect of efficacy. Further efforts should focus on improving the longitudinal behavioral knowledge of multicenter groups of f-HP patients, establishing consistent definitions of progression for use in clinical practice, and developing prognostic tools to better inform (earlier) disease progression [7].

Further research should focus on improving the behavioral understanding of large, multicenter groups of f-HP patients, developing a consensus on an agreed definition of progression for use in clinical practice and prognostic tools [8,9].

## Conclusions

HP is a form of interstitial lung disease that requires early diagnosis and treatment leading to progressive pulmonary fibrosis. Knowledge of environmental history is essential when evaluating patients with respiratory symptoms. Causes of HP include infectious agents, enzymes, animals, insects, and plant proteins, as well as low-molecular-weight chemicals and chemical compounds. Early detection is important because this condition can progress to pulmonary fibrosis, a disease associated with significant morbidity and mortality. 
